# MRI-assisted cervix cancer brachytherapy pre-planning, based on application in paracervical anaesthesia: final report

**DOI:** 10.2478/raon-2014-0009

**Published:** 2014-07-10

**Authors:** Primoz Petric, Robert Hudej, Omar Hanuna, Primoz Marolt, Noora Mohammed A A Al-Hammadi, Mohamed P. Riyas, Barbara Segedin

**Affiliations:** 1 Department of Radiation Oncology, National Center for Cancer Care and Research, Doha, Qatar; 2 Department of Radiotherapy, Institute of Oncology Ljubljana, Ljubljana, Slovenia

**Keywords:** cervix cancer, MRI, pre-planning, image-guided brachytherapy

## Abstract

**Background:**

Optimal applicator insertion is a precondition for the success of cervix cancer brachytherapy (BT). We aimed to assess feasibility and efficacy of MRI-assisted pre-planning, based on applicator insertion in para-cervical anaesthesia (PCA).

**Patients and methods.:**

Five days prior to BT, the pre-planning procedure was performed in 18 cervix cancer patients: tandem-ring applicator was inserted under PCA, pelvic MRI obtained and applicator removed. Procedure tolerability was assessed. High risk clinical target volume (HR CTV) and organs at risk were delineated on the pre-planning MRI, virtual needles placed at optimal positions, and dose planning performed. At BT, insertion was carried out in subarachnoidal anaesthesia according to pre-planned geometry. Pre-planned and actual treatment parameters were compared.

**Results:**

Pre-planning procedure was well tolerated. Median difference between the pre-planned and actual needle insertion depth and position were 2 (0–10) mm and 4 (0–30) degrees, respectively. The differences between the pre-planned and actual geometric and dosimetric parameters were statistically non-significant. All actual needles were positioned inside the HR CTV and outside the organs at risk (OAR).

**Conclusions:**

Our pre-planning approach is well tolerated and effective. Pre-planned geometry and dose distribution can be reproduced at BT.

## Introduction

MRI is the recommended modality for image guided adaptive brachytherapy (IGABT) of cervix cancer.^1^–^9^ MRI enables an accurate and reproducible delineation of the target volume and organs at risk (OAR). Individualized application techniques, treatment planning systems and remote afterloaders permit adaptation of the dose distribution to the delineated volumes and evaluation of dose volume histogram (DVH) parameters. This approach has been reflected in encouraging dosimetric and clinical results.^10^–^18^ Combined intracavitary (IC) and interstitial (IS) application techniques, using modified IC applicators are beneficial in cases of unfavourable pelvic topography and/or large tumours.^10^,^11^,^19^–^21^ At our institution, MRI-assisted IGABT, based on the GEC ESTRO recommendations, has been used since 2006.^22^–^24^ In our practice, 3D conformal external beam radiotherapy (EBRT: 45–50.4 Gy in 1.8 Gy daily fractions) +/− concurrent chemotherapy (weekly 40 mg/m2 of cisplatin) is followed by two weekly applications of MRI-assisted pulsed dose rate IGABT, prescribing a nominal dose of 18.5 Gy in 25 hourly pulses per insertion to the high risk clinical target volume (HR CTV).^17^,^18^,^25^

Accurate applicator insertion with optimal geometric distribution of eventual IS channels is a precondition for the tight control and fine-tuning of the dose distribution. The inadequacies of a suboptimal application cannot always be compensated by treatment plan optimization. Applicator insertion geometry is typically based on clinical and MRI findings at diagnosis and clinical findings at IGABT.^1^,^22^ Planning MRI is performed only after the insertion, limiting the ability for corrections in case of suboptimal implantation. In such cases, the inadequacies from the first procedure should be taken into account during eventual subsequent insertion(s), improving the cumulative dosimetric outcome.

Attempting to increase the likelihood of optimal implant geometry already at the first application, we developed an MRI-assisted pre-planning protocol, based on applicator insertion in paracervical anaesthesia (PCA). Our preliminary results on the efficacy of this technique were published recently.^25^ We report on the final results of our study here. Our primary objective was to quantify the geometric and dosimetric variation between the pre-planned and actual application and to assess feasibility of the pre-planning approach. Our secondary objective was to quantify the dosimetric benefit of the MR IGABT when compared with conventional point A-based treatment planning.

## Patients and methods

The study protocol was approved by the institutional and national ethics committees, multidisciplinary tumour board and the cost analysis office of the Institute of Oncology Ljubljana.

### Patients and tumours

Twenty consecutive patients with histologically verified inoperable cervix cancer, treated with curative intent, were enrolled after signing an informed consent. One patient was excluded from analysis due to non-compliance to the study protocol. In the second patient, pre-planning and inclusion in the dosimetric analysis was precluded by vasovagal syncope during PCA injection. Eighteen patients were eligible for the dosimetric analysis.

### Pre-planning applicator insertion in paracervical anaesthesia

**FIGURE 1.**
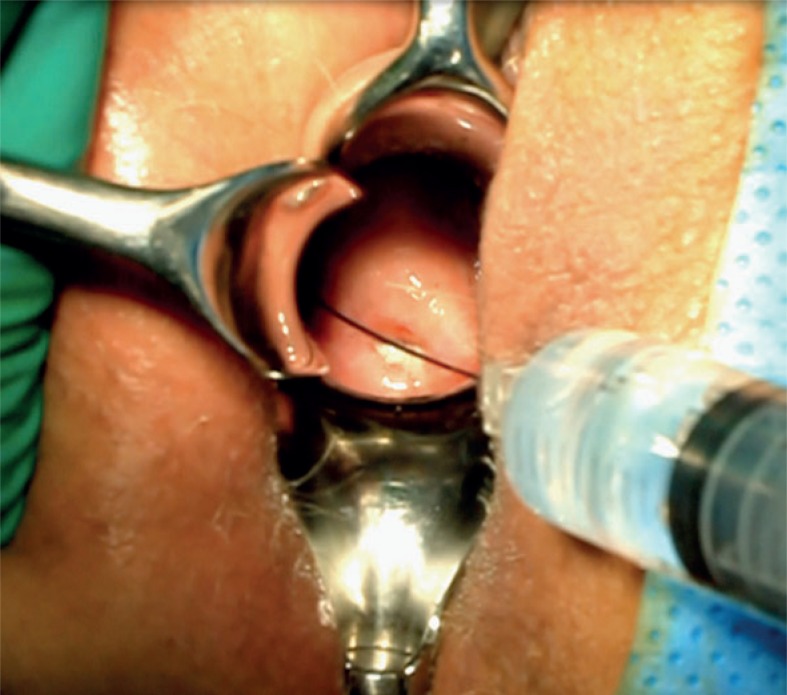
Para-cervical injection of anaesthetic prior to the pre-planning insertion of the intracavitary applicator.

Preplanning insertion was performed in sterile conditions immediately after or in the last week of EBRT. On the morning of the insertion, a laxative suppository and an anxiolytic were applied and one hour intravenous analgesic/antiemetic infusion of tramadol, metamizole and metoclopramide was administered. With the patient in lithotomy position, 10% lidocaine spray was applied topically on the vaginal mucosa and 3 mL of 2% lidocaine injected bilaterally in the paracervical region to achieve PCA (Figure 1). After 5 minutes, the anterior lip of the portio was grabbed with a tenaculum and cervical canal dilated. Following dilatation, a plastic tandem & ring applicator (©2005–2009 Varian Medical Systems, Inc., Palo Alto, USA) was inserted and vaginal packing performed. During the procedure, pain was reported by the patient, using the visual analogue scale (VAS) from 0 (no pain) to 10 (most intense pain). Peak pain score was recorded. In case of persistent pain of > 2 on VAS, additional 2 mL of 2% lidocaine was injected paracervically. In case of pain of 4 on VAS, the procedure was terminated. Patient’s vital functions were monitored and anaesthesiologist was available on call. Procedure time, from placing the patient into the lithotomy position to the applicator removal was recorded

### Pre-planning MRI and applicator removal

Pre-planning MRI was performed after applicator insertion at a 1.5 Tesla scanner (Siemens Magnetom Avanto, ©2006 Siemens AG, Erlangen, Germany), using a pelvic surface phased-array coil. 2D T2w fast spin echo images (slice thickness 3 mm, inter-slice gap 0.9 mm, in-plane pixel size 0.6 × 0.6 mm, field of view 20 × 20 cm, matrix size 288 × 320, echo time 98 ms, repetition time 5700 ms, flip angle 150°, acquisition time 3 minutes), were obtained in paratransverse (perpendicular to cervical canal) plane. In addition, a 3D T2 weighted sequence with high sampling efficiency was performed (176 slices, isotropic voxel size of 1 mm, field of view 40 x 40 cm, matrix size 384 x 386, echo time 131 ms, repetition time 1500 ms, flip angle 150 degrees, acquisition time 7 minutes). Both data sets were transferred to the treatment planning system (Brachyvision, version 8.5, Copyright ©1996–2008 Varian Medical Systems Inc., Palo Alto, USA) and co-registered, using shared DICOM coordinates. Registration was corrected manually when indicated. From the 3D data-set, para-transverse, para-coronal and para-sagittal reconstructions were resampled within the treatment planning system to match the slice thickness and acquisition planes of the 2D images.^26^ The applicator was removed after imaging and the patient discharged.

### Contouring and creation of pre-plan

**FIGURE 2.**
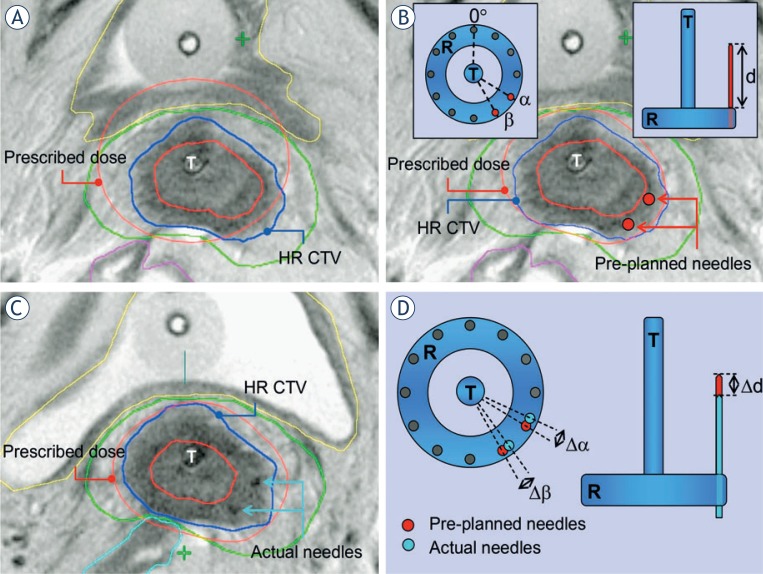
T2-weighted post-insertion pelvic MRI in para-transverse (perpendicular to cervical canal) orientation at pre-planning **(A, B)** and actual **(C)** brachytherapy. Principal steps of the pre-planning process are outlined. **(A)** Pre-planning MRI, obtained after insertion of the intracavitary tandem/ring applicator in para-cervical anaesthesia. Prescribed isodose of a standard intracavitary treatment plan with dose prescription at point A is shown. There is suboptimal coverage of the high risk clinical target volume (HR CTV) with the prescribed isodose at the left posteralateral aspect in this slice. In addition, the prescribed isodose extends to the bladder, exceeding our departmental dose constraints for this organ. Reducing the tandem dwell-weight in order to spare the posterior bladder wall would further compromise the coverage of the left part of the HR CTV due to unfavourable topography between the applicator and the patho-anatomical structures. **(B)** Virtual optimized intracavitary/interstitial pre-plan. After reducing the tandem dwell weight, two virtual interstitial needles (red circles) were placed at optimal positions within the target volume, respecting the degrees of freedom, offered by the ring cap template (Figure insert). Treatment plan optimization, utilizing needle dwell positions in addition to the intracavitary component, resulted in a pre-plan with a conformal dose distribution. The prescribed isodose conformally encompasses the HR CTV while the dose constraints for the bladder and other organs are respected. Radial angle of needle insertion position is defined on para-transverse MRI for a given ring diameter as the angle between the antero-posterior patient axis and the line, connecting the centre of the tandem and the needle. **(C)** Planning MRI, acquired at time of actual brachytherapy, following insertion of a combined intracavitary/interstitial applicator. In addition to the tandem/ring applicator, two interstitial needles were inserted through the ring template, aiming at an accurate reproduction of the pre-planned insertion angles and depths. Actual needle insertion angles and depths were recorded. Treatment plan optimization resulted in an actual dose distribution, comparable to the pre-planned situation. **(D)** Schematic representation of assessment of the geometric deviations between the pre-planned and actual implant. For each needle, the difference between the pre-planned and actual radial angle of needle insertion and depth was calculated.

HR CTV and the OAR (bladder, rectum and sigmoid colon) were delineated and the applicator reconstructed. The preplanning process started by using the standard IC loading pattern. A nominal dose of 18.5 Gy in 25 hourly pulses was specified at point A. For treatment plan optimization and reporting, biologically equivalent doses (EQD2; linear-quadratic model; α/β= 10 Gy for the tumour and 3 Gy for the OAR; repair half-time = 1.5 hours), were used. After evaluating the dose distribution and DVH parameters, the standard IC pre-plan was modified, aiming to meet our dose constraints for the OAR (D2cc - minimal EQD2 to the most exposed 2cm^3^ of the rectum, sigmoid colon and bladder below 12.5 Gy, 12.5 Gy and 15 Gy, respectively). When HR CTV was adequately covered following this modifications (D90 > prescribed dose, V100 > 90–95%), no further action was taken and the preplan was considered optimized. When indicated, virtual interstitial channels were placed at optimal radial angles and optimal insertion depths in the parametria to improve the HR CTV coverage and/or OAR avoidance (Figure 2). The degrees of freedom, offered by our modified IC applicator, in which the ring serves as a template for guidance of parametrial needles, were respected.^11^,^12^,^25^ The pre-planned radial angles of insertion positions and insertion depths were recorded (Figure 2). Virtual needles were loaded in the treatment planning system, keeping the source dwell-times below 20% of the tandem dwell-times and aiming to achieve an optimized pre-plan (adequate HR CTV coverage, while respecting the OAR dose constraints). The DVH parameters for the HR CTV (V100, D100, D90) and OAR (D2cc) of the standard and optimized pre-plan were recorded.

### Actual BT application, imaging and treatment planning

One week after the preplanning, first actual BT application was performed in subarachnoidal anaesthesia. In addition to the IC tandem-ring applicator, IS needles were inserted through the modified ring, taking the preplanned geometry (needle insertion positions and depths) into account. T2 weighted FSE MR images in para-transverse, para-coronal and para–sagittal orientation were obtained in addition to the 3D isotropic MRI with high sampling efficiency. Contouring, applicator reconstruction and treatment planning was carried out. The actual radial angles of needle insertion positions and depths were recorded and compared with the pre-planned values (Figure 2). The Standard and optimized actual treatment plans were created and the respective DVH parameters for the HR CTV and OAR compared. The optimization index (OIN) was defined as the ratio between the D90 to the HR CTV and the D2cc to the most irradiated OAR. The OINs of the standard and optimized plans were compared. Second BT application was not the subject of this study.

### Statistical analysis

The non-parametric Wilcoxon signed-rank test for matched pairs was used to compare the treatment plan parameters. The p-value of 0.05 was used as the limit of statistical significance. Statistical program SPSS was used for statistical analysis.

## Results

The patient who developed syncope following PCA injection recovered spontaneously, rapidly and without complications. However, the pre-planning procedure was terminated and the patient was ineligible for dosimetric analysis. FIGO stage distribution of the included patients was as follows: IB1: 2, IIB: 13, IIIB: 2 and IVA: 1 patient. The median HR CTV size was 26 cm^3^ (range: 15.1 – 68.3 cm^3^).

Subjective pain sensation during the pre-planning insertion in PCA was reported by all 18 patients included in the dosimetric study. Eight (44.5%) patients reported a peak VAS score of 0 (no pain); 6 (33.5%) a peak VAS score of 1; and 4 (22%) patients a peak VAS score of 3. The median duration of the pre-planning procedure was 69 minutes (range 55 to 90 minutes).

At first BT application, a total of 55 IS needles were inserted in 18 patients (median: 3.2 needles per patient; range: 0–9) through the ring template, respecting the pre-planned specifications. The differences between the pre-planned and actual geometric radial angle of insertion and insertion depth were statistically non-significant (Table 1). All actual needles were positioned inside the HR CTV and outside the OAR. The differences between the pre-planned and actual optimized DVH parameter values were not significant (Table 1).

**FIGURE 3.**
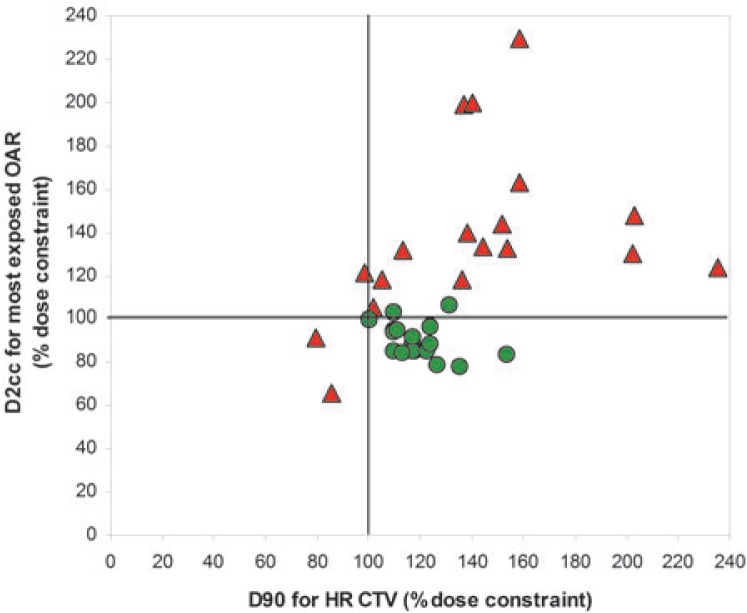
D2cc to the organ at risk (OAR) receiving the highest dose and D90 to the high risk clinical target volume (HR CTV) in standard (red triangles) and optimized (green circles) plans for all patients. The doses are expressed in percentage of respective dose constraints. The lower right quadrant includes the patients in whom both the OAR and the HR CTV dose constraints were respected.

All patients benefited from 3D MRI-guided treatment plan optimization (Table 2, Figure 3). In standard point A-based plans, the dose constraints for the D2cc to the most irradiated OAR and the D90 to the HR CTV were met in none of the 18 patients. In optimized treatment plans, both constraints were met in 16 (89%) cases. The OIN was above 1.0 in 8 (44%) and 18 (100%) patients for the standard and optimized plans, respectively. Median OIN was significantly higher for the optimized (1.3; range: 1.0–1.8), when compared with standard plans (1.0; range: 0.7–1.9) (Table 2).

## Discussion

Several reports have demonstrated favourable results of cervix cancer IGABT when compared to conventional x-ray based method.^13^–^18^,^26^ In our experience, one of the most challenging aspects of IGABT remains the decision on the optimal geometry of insertion of the applicators, which is a precondition for treatment success. The possibility to compensate for geometric inadequacies of the insertion by treatment plan optimization is limited. While there is paucity of published reports on the actual gynaecological BT pre-planning, several approaches to image guidance have been suggested to increase the chance of optimal implant geometry.^25^,^27^

US-guidance has proven helpful in achieving good position of the intrauterine tandem and is a promising method in interstitial gynaecological BT.^28^–^33^ However; adaptations of US devices and development of an US-based target concept are required before this approach can be fully exploited in practice. Currently, MRI remains the gold standard imaging modality for cervix cancer IGABT. Recommendations on different aspects of its implementation, including a target volume concept, were published.^22^–^24^,^34^ Accordingly, various methods of MRI guided applicator insertion have been proposed. One approach, which is utilized at author’s institutions in selected cases, consists of temporal interruptions of the application in order to acquire MRI for verification and off-line guidance. This, however, results in a considerable prolongation of the application time. Specialized MRI devices have been developed to allow for real-time imaging of the insertion and enable guidance in BT of different tumour sites.^35^–^39^ In one study, intra-operative MRI-guided needle insertion for BT of vaginal recurrence in endome-trial cancer demonstrated high accuracy of needle placement and limited toxicity.^35^ However, access to MRI in the operating theatre is a pre-requisite for this approach, limiting it to a few specialized centres. Alternatively, an additional MRI before BT application may identify the extent of residual disease and enable to plan an optimal insertion. Translation of pre-insertion MRI findings to the post-insertion situation is hindered by topographical changes in the pelvis, induced by the applicator insertion at BT. Ideally; the pre-planning MRI should therefore be obtained with the IC applicator in place. Due to its risks and infrastructural requirements, the need for general or spinal anaesthesia at pre-planning insertion is an important limitation to such an approach.^27^ The technique described in our study mitigates this limitation by using PCA for pain control.

PCA is used for cervical dilatation in various obstetric and gynaecological procedures. Nevertheless, the data on its effectiveness and safety are conflicting. A recent review concluded that PCA is ineffective in achieving pain control for women undergoing uterine interventions.^40^ However, in this review; various procedures including suction termination of pregnancy and bimanual removal of placenta were assessed. Therefore, its negative conclusion may not be applicable to cervical dilatation at BT. To our knowledge, there are no studies examining the role of PCA in the field of BT. A recent meta-analysis, comparing effectiveness of various local anaesthetic techniques used during outpatient hysteroscopy (which may be more comparable to uterine insertion of BT applicator), concluded that PCA is the best method of pain control in this setting.^41^ In our study, 14 (78%) patients reported a peak VAS score of 0 or 1 during the pre-planning procedure. Our favourable results are in line with conclusions of the meta-analysis. In our protocol, mild sedation was instituted prior to the procedure and local an-aesthetic applied topically to ameliorate pain during PCA injection, manipulation with specula and vaginal gauze packing. In addition, intravenous infusion of analgesics was given to reduce pain due to vaginal packing and applicator removal. We can assume that these additional measures had an important effect on the overall favourable level of pain control in our study.

With exception of one case of syncope after PCA injection (resolving spontaneously without consequences, but leading to procedure termination and exclusion from dosimetric analysis), we observed no adverse events of the pre-planning procedure. About 20% of women undergoing out-patient dilatation of cervical canal are reported to experience vasovagal reactions.^42^ It has to be noted that similar symptoms might arise from intravasation of the local anaesthetic. Meta-analysis of the studies on PCA in hysteroscopy failed to conclude on potential harms of the PCA, since most of the analysed studies did not explicitly report on the adverse events.^42^ According to our experience, IC applicator insertion under PCA can be regarded a safe procedure. This pain control technique may therefore represent a strategy to improve patient care in centres where general or spinal anaesthesia is not readily accessible. Moreover, PCA-based BT application has been used with success at our institution in patients with medical contraindications for anaesthesia, not only in cervical, but also in endometrial cancer, where co-morbidity often poses significant challenge to the medical team. It has to be noted, however, that we only assessed its effectiveness in insertion of the IC applicator, not the IS needles.

In our study, an excellent reproduction of the pre-planned implant geometry was achieved at first actual BT application, resulting in clinically and statistically non-significant dosimetric differences between the pre-plan and actual BT plan (Table 1). At actual BT, the dose constraints for the HR CTV could be respected in all cases, while they were only slightly violated for the OAR in 2 cases (2% and 6% above the constraint for the sigmoid colon and bladder, respectively) (Figure 3). As far as the benefit from pre-planning is concerned, our cohort could be divided into 2 groups of patients. In 4 (22%) patients (median HR CTV: 20 cm^3^, range: 15.1 – 26.1 cm^3^), the IS needles were not used. In these cases, the DVH constraints could be met by adapting the standard IC plan and the dosimetric benefit of pre-panning was small. In the remaining 14 patients (median HR CTV: 28.8 cm^3^, range: 17.0 – 68.3 cm^3^), the IS needles were used in addition to the IC applicator and the dosimetric benefit of pre-planning was demonstrated. To summarize, the pre-planning procedure, as described here, may be advantageous in cases with bad tumour response to EBRT and/or where unfavourable topography of residual pathological tissues at time of BT can be expected. In small and/or well responding tumours pre-planning was not beneficial.

Limitations of our pre-planning approach include the need for an additional MRI study, staff requirements and administration of additional medications. Therefore, it may be limited to centres with high resources. However, according to our experience, the pre-planning reduced the time needed for the actual BT application and improved the operator confidence and patient compliance during the procedure. In addition, the need for iterative imaging and implant corrections or even removing the applicator due to a potentially suboptimal geometry could be avoided in all cases. By omitting multi-planar MRI and employing 3D MRI for pre-planning, total image acquisition time was shortened for approximately 50%, when compared to our standard MRI study at time of actual BT.^43^ In fact, due to the possibility to achieve an optimal implant already at the first application, the described procedure may serve as a basis for accomplishment of BT in a reduced number of optimized insertions, reducing the total MRI-time and costs. The clinical impact of favourable dosimetric results of IGABT in our patient population remains to be quantified and compared with our retrospective series on conventional 2D BT.^44^

## Conclusions

MRI-assisted cervix cancer BT pre-planning, based on applicator insertion in PCA is well tolerated, safe and fast. In cases with bad tumour response to EBRT and/or unfavourable target volume topography at time of BT it enables determination of an optimal distribution of the interstitial applicator channels. At actual BT implantation, an excellent reproduction of the pre-planned insertion geometry and DVH outcome can be achieved. MR assisted IGABT improves dosimetric outcome when compared with conventional dose prescription methods. PCA may be an effective method of pain control in a setting with limited resources and in patients with medical contraindications for general or spinal anaesthesia, requiring cervico-uterine BT.

## Figures and Tables

**TABLE 1.** Needle geometry and DVH parameters of the pre-planned and actual optimized treatment plans and the individual differences between them. Biologically equivalent doses are given (linear quadratic model, α/β=10 Gy for the HR CTV and 3 Gy for the organs at risk, half time of sublethal damage repair = 1.5 h). The differences between the pre-planned and actual geometric and dosimetric parameters were statistically non-significant. HR CTV = High Risk Clinical Target Volume

	**Pre-planned median (range)**	**Actual median (range)**	**Difference median (range)**
**Needle geometry**			
Depth (mm)	23 (10 – 49)	23 (7 – 47)	2 (0 – 10)
Radial angle (°)	150 (30 – 330)	145 (30 – 334)	4 (0 – 30)
**HR CTV**			
D90 (Gy)	23.4 (20.0 – 27.1)	23.4 (20.1 – 30.7)	1.0 (0.0 – 3.6)
D100 (Gy)	13.2 (7.1 – 17.8)	14.9 (8.6 – 18.3)	1.9 (0.2 – 5.8)
V100 (%)	96.2 (90.0 – 99.8)	97.9 (90.0 – 100)	1.4 (0.1 – 9.2)
**Organs at risk**			
D2cc bladder (Gy)	13.5 (9.1 – 16.6)	12.9 (7.4 – 15.9)	0.7 (0.0 – 7.3)
D2cc rectum (Gy)	9.5 (4.9 – 15.8)	8.1 (4.2 – 11.8)	1.4 (0.3 – 11.5)
D2cc sigmoid (Gy)	10.1 (4.6 – 13.4)	9.2 (3.0 – 12.8)	1.2 (0.0 – 5.5)

**TABLE 2.** DVH parameter values for the high risk clinical target volume (HR CTV) and the most exposed organ at risk. OIN = optimization index (D90 for the HR CTV / D2cc of the OAR max).

	**Standard plan [median (range)]**	**Optimized plan [median (range)]**
**HR CTV**		
D90 (Gy)	27.9 (15.9 – 47.0)	23.4 (20.1 – 30.7)
D100 (Gy)	15.0 (7.2 – 25.7)	14.9 (8.6 – 18.3)
V100 (%)	99.0 (77.3 – 100.0)	97.9 (90.0 – 100)
**OAR_max_**		
D2cc (Gy)	19.0 (8.2 – 34.4)	12.7 (9.0 – 15.9)
**OIN**	1.0 (0.7 – 1.9)	1.3 (1.0 – 1.8)

## References

[1] Haie-Meder C, Pötter R, Van Limbergen E, Briot E, De Brabandere M, Dimopoulos J (2005). Recommendations from Gynaecological (GYN) GECESTRO Working Group (I): concepts and terms in 3D image based 3D treatment planning in cervix cancer brachytherapy with emphasis on MRI assessment of GTV and CTV. Radiother Oncol.

[2] Dimopoulous JCA, Schard G, Berger D, Lang S, Goldner G, Helbich T (2006). Systematic evaluation of MRI findings in different stages of treatment of cervical cancer: potential of MRI on delineation of target patho-anatomical structures and organs at risk. Int J Radiat Oncol Biol Phys.

[3] Boss EA, Barentsz JO, Massuger LF, Boonstra H (2000). The role of MI imaging in invasive cervical carcinoma. Eur Radiol.

[4] Subak LL, Hricak H, Powell CB, Azizi L, Stern JL (1995). Cervical carcinoma: computed tomography and magnetic resonance imaging for preoperative staging. Obstet Gynecol.

[5] Mitchell DG, Snyder B, Coakley F, Reinhold C, Thomas G, Amendola M (2006). Early invasive cervical cancer: tumor delineation by magnetic resonance imaging, computed tomography, and clinical examination, verified by pathologic results, in the ACRIN 6651/GOG 183 Intergroup Study. J Clin Oncol.

[6] Oszarlak O, Tjalma W, Scheppens E, Corthouts B, Op de Beeck B, Van Marck E (2003). The correlation of preoperative CT, MR imaging, and clinical staging (FIGO) with histopathology findings in primary cervical carcinoma. Eur Radiol.

[7] Hricak H, Gatsonsis C, Coakley FV, Snyder B, Reinhold C, Schwartz LH (2007). Early invasive cervical cancer: CT and MR imaging in preoperative evaluation – ACRIN/GOG comparative study of diagnostic performance and interobserver variability. Radiology.

[8] Jung DC, Ju W, Choi HJ, Kang S, Park S, Yoo CW (2008). The validity of tumour diameter assessed by magnetic resonance imaging and gross specimen with regard to tumour volume in cervical cancer patients. Eur J Cancer.

[9] Bipat S, Glas AS, van der Velden J, Zwinderman AH, Bossuyt PM, Stoker J (2003). Computed tomography and magnetic resonance imaging in staging of uterine cervical carcinoma: a systemic review. Gynecol Oncol.

[10] Dimopoulos JCA, Schirl G, Baldinger A, Helbich TH, Pötter R (2009). MRI assessment of cervical cancer for adaptive radiotherapy. Strahlenther Onkol.

[11] Kiristis C, Lang S, Dimopoulos J, Berger D, Georg D, Pötter R (2006). The Vienna applicator for combined intracavitary and interstitial brachytherapy of cervical cancer: design, application, treatment planning, and dosimetric results. Int J Radiat Oncol Biol Phys.

[12] Dimopoulos JCA, Kiristis C, Petric P, Georg P, Lang S, Berger D (2006). The Vienna applicator for combined intracavitary and interstitial brachytherapy of cervical cancer: clinical feasibility and preliminary results. Int J Radiat Oncol Biol Phys.

[13] Pötter R, Dimopoulos J, Georg P, Lang S, Waldhäusl C, Wachter-Gerstner N (2007). Clinical impact of MRI assisted dose volume adaptation and dose escalation in brachytherapy of locally advanced cervix cancer. Radiother Oncol.

[14] Haie-Meder C (2007). MRI-based brachytherapy (BT) in the treatment of cervical cancer: experience of the Institute Gustave-Roussy. Radiother Oncol.

[15] Lindegaard JC, Tandercup K, Nielsen SK, Haack S, Gelineck J (2008). MRI-guided 3D optimization significantly improves DVH parameters of pulsed-dose-rate brachytherapy in locally advanced cervical cancer. Int J Radiat Oncol Biol Phys.

[16] De Brabandere M, Mousa AG, Nulens A, Swinnen A, Van Limbergen E (2008). Potential of dose optimization in MRI-based PDR brachytherapy of cervix carcinoma. Radiother Oncol.

[17] Petrič P, Hudej R, Šegedin B, Zobec Logar HB (2011). MRI assisted treatment planning improves the DVH parameters in cervix cancer brachytherapy. Radiother Oncol.

[18] Hudej R, Petric P, Burger J, Jarm T, Kramar P, Županič A (2007). Standard versus 3D optimized MRI-based planning for uterine cervix cancer brachyradiotherapy-the Ljubljana experience.

[19] Jürgenliemk-Schulz IM, Tersteeg RJ, Roesink JM, Bijmolt S, Nomden CN, Moerland MA (2009). MRI-guided treatment-planning optimisation in intracavitary or combined intracavitary/interstitial PDR brachytherapy using tandem ovoid applicators in locally advanced cervical cancer. Radiother Oncol.

[20] Petrič P, Hudej R, Rogelj P, Lindegaard J, Tanderup K, Kirisits C (2010). Frequency-distribution mapping of HR CTV in cervix cancer: possibilities and limitations of existent and prototype applicators. Radiother Oncol.

[21] Kuipers T, Hoekstra CJ, van Riet A, Mak AC, Vonk EJ, Elders LH (2001). HDR brachy-therapy applied to cervical carcinoma with moderate lateral expansion: modified principles of treatment. Radiother Oncol.

[22] Pötter R, Haie-Mader C, Van Limbergen E, Barillot I, De Brabandere M, Dimopoulos J (2006). Recommendations from gynaecological (GYN) GECESTRO Working Group: (II): concepts and terms of 3D imaging, radiation physics, radiobiology, and 3D dose volume parameters. Radiother Oncol.

[23] Hellebust TP, Kirisits C, Berger D, Pérez-Calatayud J, De Brabandere M, De Leeuw A (2010). Recommendations from Gynaecological (GYN) GEC-ESTRO Working Group: Considerations and pitfalls in comissioning and applicator reconstruction in 3D image-based treatment planning of cervix cancer brachytherapy. Radiother Oncol.

[24] Dimopoulos JC, Petrow P, Tanderup K, Petric P, Berger D, Kirisits C (2012). Recommendations from Gynaecological (GYN) GEC-ESTRO Working Group (IV): Basic principles and parameters for MRI imaging within the frame of image based adaptive cervix cancer brachytherapy. Radiother Oncol.

[25] Petric P, Hudej R, Music M (2009). MRI assisted cervix cancer brachytherapy pre-planning, based on insertion of the applicator in para-cervical anaesthesia: preliminary results of a prospective study. J Contemp Brachyther.

[26] Gerbaulet A, Pötter R, Haie-Meder C, Gerbaulet A, Pötter R, Mazeron JJ, Meertens H, Van Limbergen E (2002). Cervix cancer. The GEC ESTRO Handbook of Brachytherapy.

[27] Fokdal L, Tanderup K, Hokland SB, Røhl L, Pedersen EM, Nielsen SK (2013). Clinical feasibilty of combined intracavitary/interstitial brachytherapy in locally advanced cervical cancer employing MRI with a tandem/ring applicator in situ and virtual preplanning of the interstitial component. Radiother Oncol.

[28] Granai CO, Allee P, Doherty F, Madoc-Jones H, Curry SL (1984). Ultrasound used for assessing the in situ position of intrauterine tandems. Gynecol Oncol.

[29] Sahinler I, Cepni I, Colpan D, Cepni K, Koksal S, Koca A (2004). Tandem application with transvaginal ultrasound guidance. Int J Radiat Oncol Biol Phys.

[30] Mayr NA, Montebello JF, Sorosky JI, Daugherty JS, Nguyen DL, Mardirossian G (2005). Brachytherapy management of the retroverted uterus using ultrasound-guided implant applicator placement. Brachytherapy.

[31] Davidson MT, Yuen J, D’Souza D, Radwan JS, Hammond JA, Batchelar DL (2008). Optimization of high-dose-rate cervix brachytherapy applicator placement: the benefits of intraoperative ultrasound guidance. Brachytherapy.

[32] Stock RG, Chan K, Terk M, Dewyngaert JK, Stone NN, Dottino P (1997). A new technique for performing Syed –Neblett template interstitial implants for gynecologic malignancies using transrectal-ultrasound guidance. Int J Radiat Oncol Biol Phys.

[33] Weitmann HD, Knocke TH, Waldhäusl C, Pötter R (2006). Ultrasound-guided interstitial Brachytherapy in the treatment of advanced vaginal recurences from cervical and endometrial carcinoma. Strahlenther Onkol.

[34] Petric P, Pötter R, Van Limbergen E, Haie Meder C, Viswanathan AN (2011). Adaptive contouring of the target volume and organs at risk. Gynecologic radiation therapy: novel approaches to image-guidance and management.

[35] Viswanathan AN, Cormack R, Holloway CL, Tanaka C, O’Farrell D, Devlin PM (2006). Magnetic resonance-guided interstitial therapy for vaginal recurrence of endometrial cancer. Int J Radiat Oncol Biol Phys.

[36] D’Amico AV, Cormack R, Tempany CM, Kumar S, Topulos G, Kooy HM (1998). Real-time magnetic resonance image-guided interstitial brachytherapy in the treatment of select patients with clinically localized prostate cancer. Int J Radiat Oncol Biol Phys.

[37] Menard C, Susil RC, Choyke P, Gustafson GS, Kammerer W, Ning H (2004). MRI-guided HDR prostate brachytherapy in standard 1.5T scanner. Int J Radiat Oncol Biol Phys.

[38] Albert M, Tempany CM, Schultz D, Chen MH, Cormack RA, Kumar S (2003). Late genitourinary and gastrointestinal toxicity after magnetic resonance image-guided prostate brachytherapy with or without neoadjuvant external beam radiation therapy. Cancer.

[39] Kettenbach J, Pokrajac B, Schamp S, Fellner C, Schmid R, Gustorff B (2001). MRI-assisted brachytherapy of nonresectable liver metastases. Preliminary technical and clinical experiences. Radiologe.

[40] Tangsiriwatthana T, Sangkomkamhang US, Lumbiganon P, Laopaiboon M (2013). Paracervical local anaesthesia for cervical dilatation and uterine intervention. Cochrane Database of Systematic Reviews.

[41] Cooper NA, Khan KS, Clark TJ (2010). Local anaesthesia for pain control during outpatient hysteroscopy: systematic review and meta-analysis. Br Med J.

[42] Finikiotis G (1993). Side-effects and complications of outpatient hysteroscopy. Aust N Z J Obstet Gynaecol.

[43] Petric P, Hudej R, Rogelj P, Blas M, Segedin B, Logar HB (2012). Comparison of 3D MRI with high sampling efficiency and 2D multiplanar MRI for contouring in cervix cancer brachytherapy. Radiol Oncol.

[44] Zobec Logar HL, Segedin B, Hudej R, Petric P (2013). Definitive radiotherapy for uterine cervix cancer: long term results for patients treated in the period from 1998 till 2002 at the Institute of Oncology Ljubljana. Radiol Oncol.

